# Quantum super-resolution imaging: a review and perspective

**DOI:** 10.1515/nanoph-2024-0597

**Published:** 2025-01-16

**Authors:** Xiaoran Yue, Hui Wu, Jizhou Wang, Zhe He

**Affiliations:** Shandong Institute of Advanced Technology, Jinan, Shandong 250000, China; University of Rochester, Rochester, NY 14620, USA; Texas A&M University, College Station, TX 77840, USA

**Keywords:** quantum imaging, super-resolution imaging, quantum entanglement

## Abstract

Quantum super-resolution imaging provides a nonlabeling method to surpass the diffraction limit of imaging systems. This technique relies on measurement of the second-order correlation function and usually employs spatially entangled photon sources. We introduce recent methods that achieve spatial resolution enhancement through quantum approaches, particularly the imaging techniques utilizing biphoton states. The fundamental mechanisms are discussed in detail to explain why biphoton states enable super-resolution. Additionally, we introduce multiple algorithms that extract the correlation function from the readings of two-dimensional detectors. Several cases are reviewed to evaluate the advantages and prospects of quantum imaging, along with a discussion of practical developments and potential applications.

## 1 Introduction

Optical microscopy is designed to observe small objects that cannot be seen by naked eyes. Due to the wave-like nature of light, phenomena such as diffraction and interference hinder the formation of a perfect image, even when the optical system is optimal [1], [2], [3]. Consequently, spatial resolution becomes an important parameter to evaluate the imaging system, defined as the minimum distance between two-point sources at which they can be distinguished as separate entities [4], [5]. The spatial resolution of an ideal imaging system is fundamentally limited by the physics of diffraction and can be estimated by the Abbe diffraction limit *λ*/2NA, where *λ* is the light wavelength, and NA represents the numerical aperture of the system [6], [7].

Advancements in optical imaging have demonstrated that spatial resolution can exceed the diffraction limit, a phenomenon known as super-resolution [8]. This development has led to substantial breakthroughs in super-resolution microscopy, which is prominently represented by techniques including stimulated emission depletion (STED) microscopy [9], [10], [11], [12], structured illumination microscopy (SIM) [13], [14], [15], [16], and stochastic optical reconstruction microscopy (STORM) [17], [18], [19].

In STED microscopy, two laser beams are usually employed to create a donut-shaped depletion laser surrounding the focal spot of an excitation laser. The first laser excites the fluorophores, while the depleting laser depletes the emission of unwanted fluorescence at the periphery, thereby preventing it from contributing to the final image. This targeted depletion reduces the point spread function (PSF), allowing for more precise focusing of light onto a smaller region on an object, achieving imaging down to 20–50 nm [20], [21], [22]. In SIM, the sample is illuminated with patterned light stripes at varying orientations and positions. Applying Fourier transform analysis to the resulting images, a higher-resolution image can be reconstructed. The resolution can be improved to be half of the diffraction limit [1], [23]. STORM works by selectively exciting a small fraction of fluorophores at any given moment, thereby preventing the overlap of emissions that would otherwise cause blurring. By capturing sequential images containing emissions from a small number of fluorophores in each frame, a composite super-resolution image can be reconstructed. While these classical super-resolution techniques can effectively improve spatial resolution, they either require fluorescent labeling or have slow imaging speed.

Recently, quantum super-resolution has emerged as a novel method for super-resolution imaging without labeling. Unlike classical super-resolution techniques that measure the first-order intensity, quantum imaging is usually based on the second-order correlation function [24]. Here, an example is quantum image scanning microscopy (Q-ISM), which combines quantum correlations with image scanning microscopy (ISM) to achieve resolutions up to twofold beyond the diffraction limit without compromising signal strength [25]. Measuring the two-photon correlation and using the confocal configuration enhance the original PSF by a factor of $\sqrt{2}$ , respectively. Hence, combining both enhancement effects, the effective PSF of this technique is narrowed by a factor of two to the original PSF. However, the drawback of Q-ISM is the long acquisition time for a high signal-to-noise ratio (SNR) because of the low counting rate of two-photon detection events, which can be improved by using entangled photon source [26], [27], [28].

Another type of quantum imaging is quantum microscopy by coincidence (QMC) [29], which exploits spatial entanglement – a phenomenon in which particles remain correlated regardless of spatial separation [30], [31], [32]. Here, the two entangled photons are termed as the signal and idler photons, respectively. Through biphoton detection, this method improves spatial resolution by halving the effective wavelength [29], [33]. Therefore, the spatial resolution reaches half of that given by the similar classical imaging methods. Besides resolution enhancement, quantum imaging with entangled sources usually presents a higher SNR by selecting only correlated photon coincidences, which excludes stray light and uncorrelated background noise [34], [35], [36]. However, this technique also operates at a slower speed compared to classical imaging systems as it requires multiple frames to statistically evaluate the correlation between signal and idler images over time.

At the current stage, quantum imaging is challenged by the complex setups and slower speed compared to classical optical imaging techniques. However, the unique advantages afforded by entangled photon states apply quantum imaging for scenarios beyond the capability of classical methods. Specifically, measuring the second-order correlation of entangled photons enables quantum imaging to work with an extremely low light field. The detection approaches effectively suppress uncorrelated background noise and achieve sub-shot-noise imaging, which is inherently nonclassical in nature. In contrast, classical imaging SNRs are limited by both environmental noise and shot noise, especially in situations requiring single-photon detection, such as cryogenic environments. Quantum imaging also benefits from the special properties of entanglement. For example, polarization entanglement allows for phase and dark-field imaging through nonlocal operations, utilizing the nonlocality of quantum entanglement. Spatially entangled photons can be used for ghost imaging without spatially resolvable cameras behind the target, valuable in extreme condition where the camera is easily damaged. Besides, quantum super-resolution also serves as a unique application based on the spatial entanglement of photons.

In this review, we will discuss the fundamental mechanisms of quantum super-resolution imaging and compare various algorithms for wide-field quantum imaging. Several types of wide-field quantum imaging techniques will be introduced, highlighting their innovative methods. We then analyze the feasibility of realizing quantum super-resolution using raster scanning microscopy, specifically clarifying the resolution enhancement achieved through biphoton measurement and the entanglement pinhole effect. Finally, we outline future development opportunities within this field.

## 2 Fundamentals

### 2.1 Spatial entanglement

Spatial entanglement is a quantum phenomenon where the wavefunctions related to spatial properties of particles are correlated, even when they are spatially separated. This type of entanglement is usually generated through a process known as spontaneous parametric down-conversion (SPDC) [37], [38]. SPDC occurs when a nonlinear crystal converts a single high-energy photon into two lower-energy photons.

The quantum state of these entangled photons can be represented by the wavefunction $\Psi \left(r_{s},r_{i}\right)$ , which represents the probability of finding the signal photon at position ***r***_*s*_ and the idler photon at position ***r***_*i*_. In the SPDC process, high-energy pump photons with specific momentum ***k***_*p*_ and frequency *ω*_*p*_ are absorbed by a nonlinear crystal, emitting two lower-energy photons with the momentum ***k***_*s*_ and ***k***_*i*_, and frequency *ω*_*s*_ and *ω*_*i*_, respectively. Both momentum and energy are conserved in this process, referring to the phase matching condition ***k***_*p*_ = ***k***_*s*_ + ***k***_*i*_ and *ω*_*p*_ = *ω*_*s*_ + *ω*_*i*_ [39]. The spatial wave function $\Psi \left(r_{s},r_{i}\right)$ can be defined as

(1) $$\Psi \left(r_{s},r_{i}\right)=\underset{k_{s},k_{i}}{\sum }{\text{e}}^{-\text{i}k_{s}\cdot r_{s}}{\text{e}}^{-\text{i}k_{i}\cdot r_{i}}\left|k_{s},k_{i}\right\rangle ,$$

where $\left|k_{s},k_{i}\right\rangle$ represents the biphoton state in the momentum domain. This pure biphoton state is also expressed as

(2) $$\left|k_{s},k_{i}\right\rangle =a_{k_{s}}^{†}a_{k_{i}}^{†}\left|0\right\rangle .$$

$\left|0\right\rangle$ is the vacuum state. $a_{k_{s}}^{†}$ and $a_{k_{i}}^{†}$ are the creation operators. Similarly, the wavefunctions of the signal and idler photons are

(3) $$\Psi_{s}\left(r_{s}\right)=\underset{k_{s}}{\sum }{\text{e}}^{-\text{i}k_{s}\cdot r_{s}}\left|k_{s}\right\rangle ,$$

(4) $$\Psi_{i}\left(r_{s}\right)=\underset{k_{i}}{\sum }{\text{e}}^{-\text{i}k_{i}\cdot r_{i}}\left|k_{i}\right\rangle .$$

On the imaging plane, the detected image corresponding to the signal and idler photons can be observed, respectively:

(5) $$G_{s}^{\left(1\right)}\left(r_{s}\right)=\left\langle \Psi_{s}\left(r_{s}\right)\right|E_{s}^{\left(-\right)}E_{s}^{\left(+\right)}\left|\Psi_{s}\left(r_{s}\right)\right\rangle ,$$

(6) $$G_{i}^{\left(1\right)}\left(r_{i}\right)=\left\langle \Psi_{i}\left(r_{i}\right)\right|E_{i}^{\left(-\right)}E_{i}^{\left(+\right)}\left|\Psi_{i}\left(r_{i}\right)\right\rangle .$$

Quantum correlations between the entangled photons can be measured through the second-order correlation function [40], [41]:

(7) $$G^{\left(2\right)}\left(r_{s},r_{i}\right)=\left\langle \Psi \left(r_{s},r_{i}\right)\right|E_{i}^{\left(-\right)}E_{s}^{\left(-\right)}E_{s}^{\left(+\right)}E_{i}^{\left(+\right)}\left|\Psi \left(r_{s},r_{i}\right)\right\rangle .$$

We will introduce the property of biphoton sources, why $G^{\left(2\right)}$ measurement enhance the spatial resolution over the classical imaging that measures $G_{s}^{\left(1\right)}$ , and the detection methods of $G^{\left(2\right)}$ .

### 2.2 Spontaneous parametric down-conversion

Spontaneous parametric down-conversion (SPDC) is a quantum optical process that generates entangled photon pairs. These pairs can be entangled across various degrees of freedom, such as polarization, energy, and momentum [42], [43], [44], [45]. Entanglement in polarization was used for Bell’s test to verify the EPR paradox [46], [47]. Besides, it is widely used in quantum communications and quantum computing, as the horizontal $\left|H\right\rangle$ and vertical $\left|V\right\rangle$ states perform as a discrete two-level system, allowing a concise computing protocol [48], [49], [50]. The energy entanglement is usually applied to generate biphotons with different wavelengths [51]. For example, the object easily damaged by infrared light can be imaged via ghost imaging using IR and visible photons. Position-momentum entanglement or spatial entanglement will be discussed in detailed in this paper. It enhances the resolution of imaging by measuring the coincidence events of entangled photon pairs [52], [53]. A photon source with multiple types of entanglement is also known as high-dimensional entangled [54], [55], [56]. The high-dimensional entanglement can be realized using different nonlinear crystals, including beta barium borate (BBO), periodically poled potassium titanyl phosphate (PPKTP), and periodically poled lithium niobate (PPLN), which offers different types of entanglement.

BBO crystals offer a broad frequency range extending from visible to infrared, making them versatile for a variety of applications [57], [58]. One BBO crystal can generate photon pairs with correlated polarizations. When stacking two BBO crystals with perpendicular optical axis, the source generates entangled photon states, for example, the type-I SPDC state in BBO is

(8) $$\left|\Psi_{\text{BBO}}\right\rangle =\frac{1}{\sqrt{2}}\left(\left|HH\right\rangle +\left|VV\right\rangle \right).$$

However, a significant challenge with BBO is the difficulty in achieving phase matching, which requires precise angle tuning and temperature control.

PPKTP and PPLN crystals employ quasi-phase matching, enabling efficient SPDC over a wider range of wavelengths without requiring careful angle tuning. Nevertheless, they are usually applied to specific wavelengths around 810 nm and 1,550 nm, especially, PPLN is well suitable for telecom wavelength at 1,550 nm [59], [60]. These crystals generate photons with high-dimensional entanglement, providing higher stability and source power than BBO. However, all three nonlinear crystals suffer from photorefractive damage when exposed to high-intensity light.

### 2.3 Interference with entangled photons

**Figure 1:**
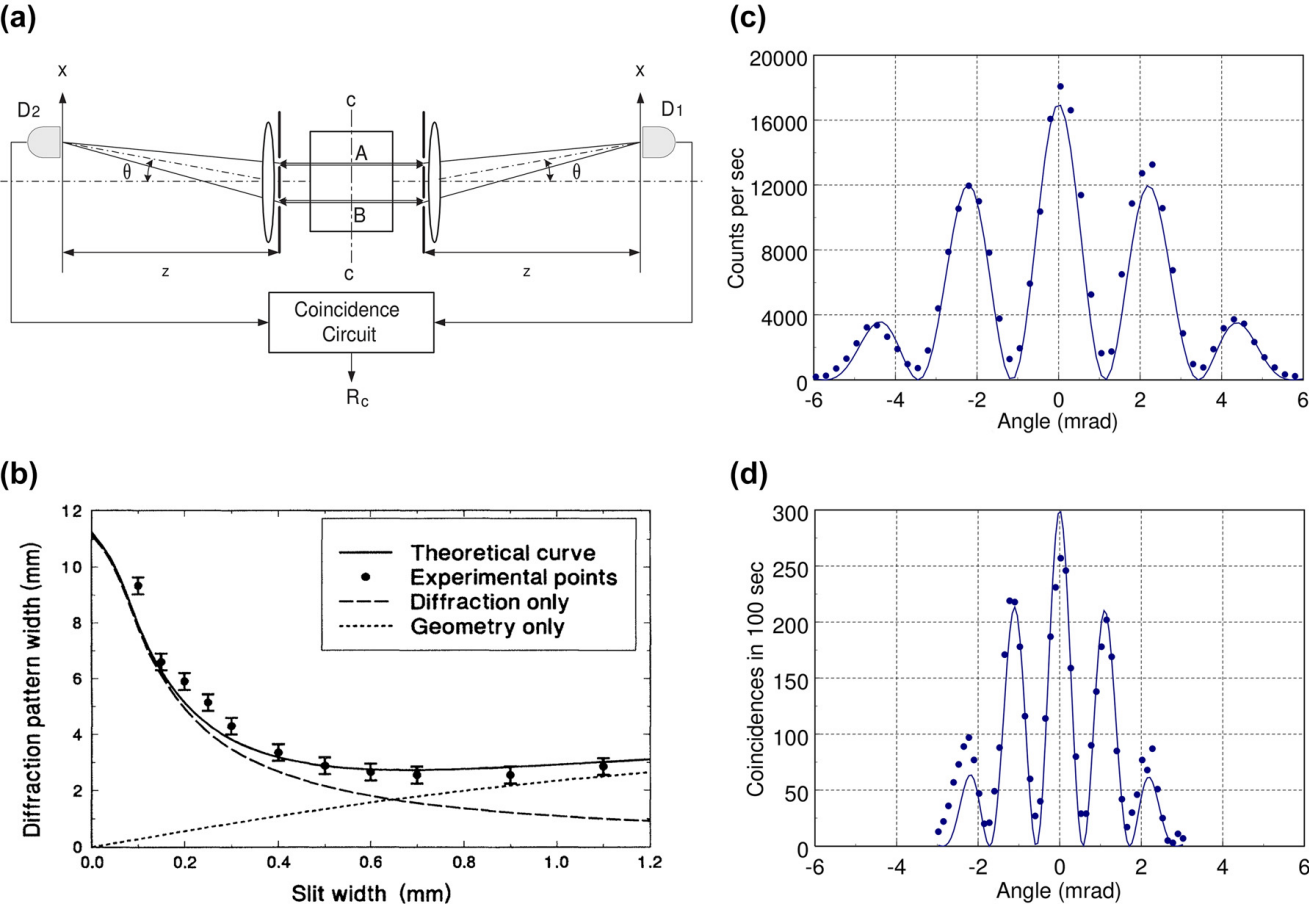
Two-photon diffraction and interference. (a) Simplified schematic of a biphoton diffraction–interference. When biphotons propagate through a double slit, the setup is equivalent to (a). D_1_ and D_2_ are the photon detectors. Their counts are sent to a coincidence circuit for $G^{\left(2\right)}$ measurement. (b) Diffraction pattern width in the biphoton diffraction experiment. The experimental result does not match the classical diffraction theory. (c) Interference pattern of classical light in the double-slit experiment. (d) Interference pattern of biphotons in the double-slit experiment [40], [41]. Copyright 2001 American Physical Society, 1995 American Physical Society.

Abbe’s theory indicates that spatial resolution is related to the wavelength of photons involved. In coincidence measurements, the effective wavelength of biphotons cannot be measured directly; rather, it can be inferred from the interference pattern. The frequency of the interference pattern in quantum imaging is twice that observed in classical imaging, as demonstrated in Figure 1(c) and (d) [41]. Given the proportional relationship between the frequency of the interference pattern and light source, Figure 1 suggests that the effective wavelength of biphotons is half that of the actual wavelength in photon pairs. This higher frequency in the quantum interference pattern correlates with an enhanced ability to resolve finer details within an image.

In the case where entangled photons are involved, super-resolution can be achieved by detecting coincidence events. Let’s assume the double slits experiment with biphotons [41]. The experiment setup is depicted in Figure 1(a), where the biophotons propagate through a double slit, and the coincidence is measured by two detectors, D1 and D2. The electric field at the detector D_1_ through the slit A and B are defined as $E_{A1}=u_{0}{\text{e}}^{-\text{i}k\cdot r_{A1}}$ and $E_{B1}=u_{0}{\text{e}}^{-\text{i}k\cdot r_{B1}}$ . And the electric field at the detector D_2_ through the slit A and B are defined as $E_{A2}=u_{0}{\text{e}}^{-\text{i}k\cdot r_{A2}}$ and $E_{B2}=u_{0}{\text{e}}^{-\text{i}k\cdot r_{B2}}$ . *u*_0_ is the amplitude of the electric field of both photons. Since we can place the double slits at symmetric positions, the wavevectors related to both slits can be simplified to be the same, ***k*** = ***k***_*A*_ = ***k***_*B*_. The classical interference intensity by D_1_ is

(9) $$\begin{array}{cc} G_{1}^{\left(1\right)} & ={\left|E_{1}\right|}^{2}={\left|E_{A1}+E_{B1}\right|}^{2}\\ & =2{\left|u_{0}\right|}^{2}\left(1+\cos\left(k\cdot \left(r_{B1}-r_{A1}\right)\right)\right). \end{array}$$

Similarly, for detector D_2_, the interference intensity is

(10) $$\begin{array}{cc} G_{2}^{\left(1\right)} & ={\left|E_{2}\right|}^{2}={\left|E_{A2}+E_{B2}\right|}^{2}\\ & =2{\left|u_{0}\right|}^{2}\left(1+\cos\left(k\cdot \left(r_{B2}-r_{A2}\right)\right)\right). \end{array}$$

Combining the results of both detectors, the second-order correlation function is measured as

(11) $$G^{\left(2\right)}=\left\langle \Psi \right|E_{1}^{\left(-\right)}E_{2}^{\left(-\right)}E_{2}^{\left(+\right)}E_{1}^{\left(+\right)}\left|\Psi \right\rangle .$$

Hence, we can define $a_{A}^{†}$ and $a_{B}^{†}$ as the creation operators of photons passing through the slits *A* and *B*, which is a reduced model of Eqs. (1) and (2). $E_{1}^{\left(+\right)}$ and $E_{2}^{\left(+\right)}$ are the quantized field. The initial phases of the wavefunction in Eq. (1) are not considered. The combined wavefunction becomes

(12) $$\left|\Psi \right\rangle =\left(a_{A,s}^{†}a_{A,i}^{†}+a_{B,s}^{†}a_{B,i}^{†}\right)\left|0\right\rangle .$$

The $G^{\left(2\right)}$ then becomes

(13) $$G^{\left(2\right)}=2{\left|u_{0}\right|}^{2}\left(1+\cos\left[k\cdot \left(r_{B1}+r_{B2}-r_{A1}-r_{A2}\right)\right]\right).$$

For entangled photons detected by two detectors aligned symmetrically, the phases ***k*** ⋅ ***r***_*B*1_ = ***k*** ⋅ ***r***_*B*2_. Therefore, $G^{\left(2\right)}$ shows a similar equation as the interference pattern of the signal or idler photon [41]:

(14) $$G^{\left(2\right)}=2{\left|u_{0}\right|}^{2}\left(1+\cos\left[2k\cdot \left(r_{B1}-r_{A1}\right)\right]\right).$$

The difference is the spatial frequency of the $G^{\left(2\right)}$ is twice of $G_{1}^{\left(1\right)}$ , which indicates that the effective wavelength of biphoton is *λ*/2. The interference patterns corresponding to $G_{1}^{\left(1\right)}$ and $G^{\left(2\right)}$ are demonstrated in Figure 1(c) and (d).

### 2.4 Spatial resolution of quantum imaging

In the case of imaging, the two-photon interference pattern can be replaced by a diffraction pattern. We assume that two entangled photons pass through a single slit with an infinitesimal width *d*. The classical diffraction field for the signal photon is given by

(15) $$E_{s}=u_{0}\underset{k_{s}}{\sum }{\text{e}}^{\text{i}k_{s}\cdot r_{s}}.$$

The summation over ***k***_*s*_ indicates all possible paths through the slit and can be replaced by an integral over the slit area *x*:

(16) $$E_{s}=u_{0}\overset{+d/2}{\underset{-d/2}{\int }}{\text{e}}^{\text{i}k_{s,⊥}x}{\text{e}}^{\text{i}\varphi_{s}}dx,$$

where *k*_*s*,⊥_ is the projection of the wavevector on the slit plane; *φ*_*s*_ is the propagation phase of the signal photon. The integral returns

(17) $$E_{s}=u_{0}d\text{sinc}\left(\frac{k_{s,⊥}d}{2}\right){\text{e}}^{\text{i}\varphi_{s}}.$$

Therefore, the diffraction pattern is

(18) $$G_{s}^{\left(1\right)}=u_{0}^{2}d^{2}\sin{\text{c}}^{2}\left(\frac{k_{s,⊥}d}{2}\right).$$

In the classical imaging system, the diffraction pattern of a slit can be used to estimate the resolution. The bandwidth of the central maximum is related to the spatial resolution.

Now we detect the entangled photons simultaneously, the quantized electric field for the signal and idler photons are

(19) $$E_{s}^{\left(+\right)}=u_{0}\underset{k_{s}}{\sum }a_{k_{s}}{\text{e}}^{\text{i}k_{s}\cdot r_{s}},$$

(20) $$E_{i}^{\left(+\right)}=u_{0}\underset{k_{i}}{\sum }a_{k_{i}}{\text{e}}^{\text{i}k_{i}\cdot r_{i}}.$$

Similar to the interference case, the wavefunction of the biphoton is simplified as

(21) $$\left|\Psi \right\rangle =\underset{k_{s}}{\sum }a_{k_{s},s}^{†}a_{k_{i},i}^{†}\left|0\right\rangle .$$

Since the wavevectors ***k***_*s*_ and ***k***_*i*_ are related, we only have to sum over ***k***_*s*_. $G^{\left(2\right)}$ becomes

(22) $$G^{\left(2\right)}={\left|u_{0}\right|}^{2}{\left|\underset{k_{s}}{\sum }{\text{e}}^{\text{i}k_{s}\cdot r_{s}}{\text{e}}^{\text{i}k_{i}\cdot r_{i}}\right|}^{2}.$$

To have a clear view of the correlation between the two photons, we have the same phases, ***k***_*s*_ ⋅ ***r***_*s*_ = ***k***_*i*_ ⋅ ***r***_*i*_:

(23) $$G^{\left(2\right)}={\left|u_{0}\right|}^{2}{\left|\underset{k_{s}}{\sum }{\text{e}}^{\text{i}2k_{s}\cdot r_{s}}\right|}^{2}.$$

We can repeat the calculations above to show that the final expression of $G^{\left(2\right)}$ is similar to that of $G_{s}^{\left(1\right)}$ , given by [41]

(24) $$G^{\left(2\right)}=u_{0}^{2}d^{2}\sin{\text{c}}^{2}\left(k_{s,⊥}d\right).$$

Here, $G^{\left(2\right)}$ is a sinc function as well. The bandwidth of the central pattern is half that of $G_{s}^{\left(1\right)}$ . Because the phases of entangled photons are correlated, when it comes to the spatial performance, biphotons can be treated as one with a double spatial frequency, or with a half wavelength. For this reason, compared to the signal or idler photon, the resolution of biphoton achieves super-resolution at the Heisenberg limit [29], [61].

## Method of $G^{\left(2\right)}$ imaging

3

The standard $G^{\left(2\right)}$ measurement of entangled photons involves two single-photon detectors [62]. These detectors are connected to a time-correlated single-photon counting module (TCSPC) to record coincidence events within a specific timeframe, typically less than 10 ns [57], [58]. Important features of $G^{\left(2\right)}$ measurement include the sensitivity, temporal resolution, and quantum efficiency of detectors. High sensitivity and temporal resolution are important for effectively capturing single photons. High quantum efficiency (QE) is necessary to ensure paired photons are simultaneously detected. Otherwise, the true coincidence may be low even with a large raw count, and accidental coincidences can degrade the SNRs.

Two-dimensional cameras, such as EMCCDs, offer sensitivity for single-photon counting and high quantum efficiency of over 80 % [63]. However, the frame rates of these cameras are typically low [63], [64]. This means that the signal detected by any single pixel may represent the accumulation of multiple photons over time, preventing accurate measurement of the true coincidence rate. To address this issue, various algorithms have been developed.

The simplest algorithm mimics the TCSPC process. For example, with an EMCCD at a frame rate of 100 Hz, the biphoton events per pixel must remain below 100 Hz to ensure each pixel contains at most one photon pair. The EMCCD readings can be transformed into photon-counting events by adding a proper threshold. The threshold is typically chosen as the mean value plus three times the standard deviation of the background. Correlated pixels are matched using an “AND” gate to identify coincidence events per pixel [34]. If no ambiguous events arise, this algorithm is theoretically efficient. However, EMCCDs, typically operated at temperatures above −90 °C, may not guarantee expected frames. Noise and unexpected rays introduce errors that significantly impact measurement under low-intensity field. To maintain high SNRs, coincidence imaging needs a large number of accumulated frames to statistically reduce the noise [29], [33], [34]. In the following section, we will discuss experimental approaches to quantum super-resolution imaging. This includes the development of various algorithms designed to enhance the performance of the “AND” algorithm.

## 4 Approaches to quantum super-resolution imaging

### 4.1 Wide-field quantum imaging

Wide-field quantum imaging typically employs two-dimensional cameras, such as EMCCDs, sCMOSs, and SPADs, to capture detailed images. In conventional systems, a multimode biphoton beam is used to illuminate the object, and the transmission light carries various information based on the types of entanglement. For example, a polarization-entangled biphoton source can extract phase information from an object, even when the light is incoherent [33]. Additionally, polarization entanglement is utilized in birefringence ghost imaging, a method that differs from traditional approaches and does not require paired polarizers in front of and behind the object [65].

The quantum source generates beams that are multimode in both spatial and frequency domains. In the spatial domain, the multimode beam refers to a continuous variable of wavevectors. As indicated by the wavefunction equations, the wavefunction of this beam involves a summation over ***k***. Consequently, neither the signal nor the idler beam can be focused to the size of a single-mode light, making it suitable for wide-field imaging.

In the wide-field imaging configuration, spatial resolution is determined by both the pixel size and the numerical aperture (NA) of the system. If the pixel size of the 2D camera is significantly smaller than the diffraction limit, the resolution of conventional wide-field imaging can be estimated as *λ*/2NA.

A comprehensive interpretation of quantum super-resolution in this case is that by detecting correlated idler photons, specific signal photons are selected to image the object. These selected signal photons show the performance of biphoton. The function of biphoton coincidence, as interpreted theoretically, is similar to that of single-photon counting, but with half wavelengths. This differs from classical methods such as confocal microscopy. In confocal microscopy, signal photons are selected based on both the source and detection sides, resulting in a resolution improvement by $\sqrt{2}$ . The confocal mechanism is well-explained using the wave model of light [66], [67]. However, quantum imaging suggests that two photons can behave as one with different frequencies. This phenomenon is determined by the measurement of the second-order correlation function and the unique properties of the entangled state, which cannot be explained using a classical imaging model.

As an example of quantum super-resolution at the Heisenberg limit [35], [68], QMC employs a wide-field illumination configuration. In this setup, a hemisphere-shaped light source illuminates the object on the signal arm, while a symmetric imaging setup is employed on the idler arm without an object. QMC simultaneously captures images from both the signal and idler arms using an EMCCD camera and reconstructs the final image based on the second-order correlation.

**Figure 2:**
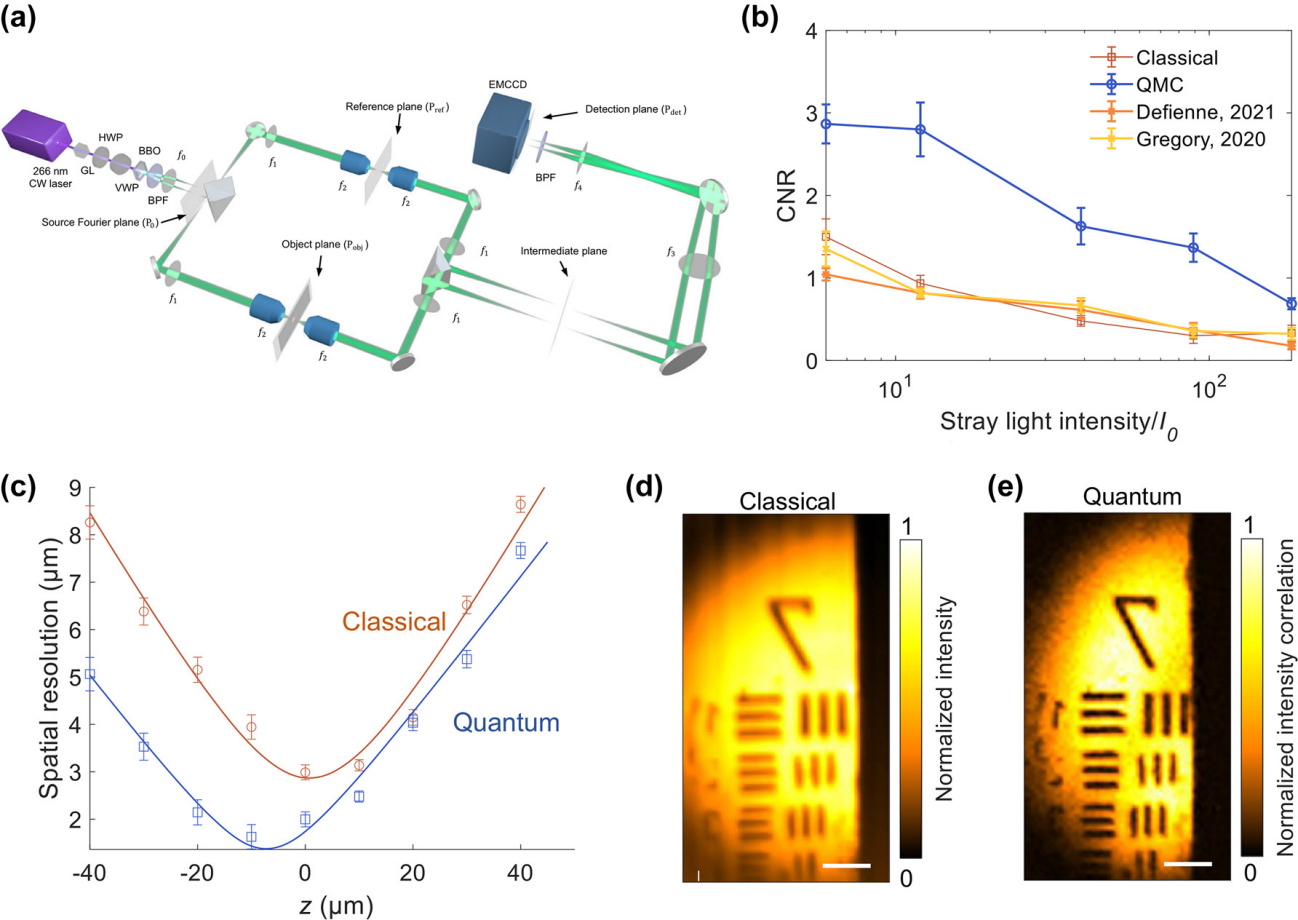
Quantum microscopy by coincidence (QMC). (a) Experimental setup of QMC. The signal beam illuminates the object positioned at the object plane. Both arms are symmetry to ensure the path length of the signal and idler photons are the same. (b) The contrast-to-noise ratios (CNRs) using different methods are evaluated over 10^5^ frames in the presence of stray light. (c) The spatial resolution of classical imaging compared to QMC in relation to the axial *z* coordinate from the classical focal point. The highest spatial resolutions achieved are 2.9 μm for classical imaging and 1.4 μm for QMC, respectively. Classical imaging (d) and QMC (e) show group 7 (2.76–3.91 μm) of a USAF 1951 resolution target. The images include scale bars of 20 μm and have been normalized [29]. Copyright 2022 Springer Nature.

In terms of resolution, the experiment achieves a spatial resolution of approximately 1.4 µm, compared to 2.8 µm achieved by classical methods, as depicted in Figure 2(c) [33]. This graph illustrates the resolution near the focal point for both classical and quantum imaging. We note that Figure 2(c) demonstrates that the focal positions of the signal photon and biphoton beams are different, which still remains unexplained.

Furthermore, as an improvement to the “AND” algorithm introduced in the previous section, covariance is used to represent the coincidence [29]. Let the readings of one frame be *I*_*s*_ and *I*_*i*_ for the signal and idler beams, respectively. The noise readings are denoted as $I_{s}^{\text{noise}}$ and $I_{i}^{\text{noise}}$ . The goal is to determine the expected value of the coincidence reads *I*_*c*_. The covariance is given by

(25) $$\text{cov}\left(I_{s},I_{i}\right)=\frac{1}{N}\overset{N}{\underset{j}{\sum }}\left(I_{s,j}-{\bar{I}}_{s,j}\right)\left(I_{i,j}-{\bar{I}}_{i,j}\right).$$

Readings in the *j*’s frame *I*_*s*,*j*_ and *I*_*i*,*j*_ are given by

(26) $$I_{s,j}=I_{c,j}+I_{s,j}^{\text{noise}},$$

(27) $$I_{i,j}=I_{c,j}+I_{i,j}^{\text{noise}}.$$

Substitute the variables above:

(28) $$\text{cov}\left(I_{s},I_{i}\right)=\bar{I_{c}^{2}}-{\bar{I}}_{c}^{2}+\bar{I_{s}^{\text{noise}}I_{i}^{\text{noise}}}-\bar{I_{s}^{\text{noise}}}\cdot \bar{I_{i}^{\text{noise}}}.$$

If the variance of *I*_*c*_ is much larger than the covariance of the noise, the covariance becomes

(29) $$\text{cov}\left(I_{s},I_{i}\right)=\bar{I_{c}^{2}}-{\bar{I}}_{c}^{2}.$$

The coincidence counting has been experimentally proved to follow a Poisson distribution [65]; therefore, the covariance of the raw count equals the coincidence count:

(30) $$\text{cov}\left(I_{s},I_{i}\right)={\bar{I}}_{c}.$$

The covariance algorithm exploits the fact that noise in the two detection areas for paired photons is uncorrelated, allowing it to filter out noise and accidental coincidences. Compared to the “AND” algorithm, which eliminates noise through thresholding, the covariance method uses the different physical properties of the true coincidence and noise, making it more accurate and effective.

**Figure 3:**
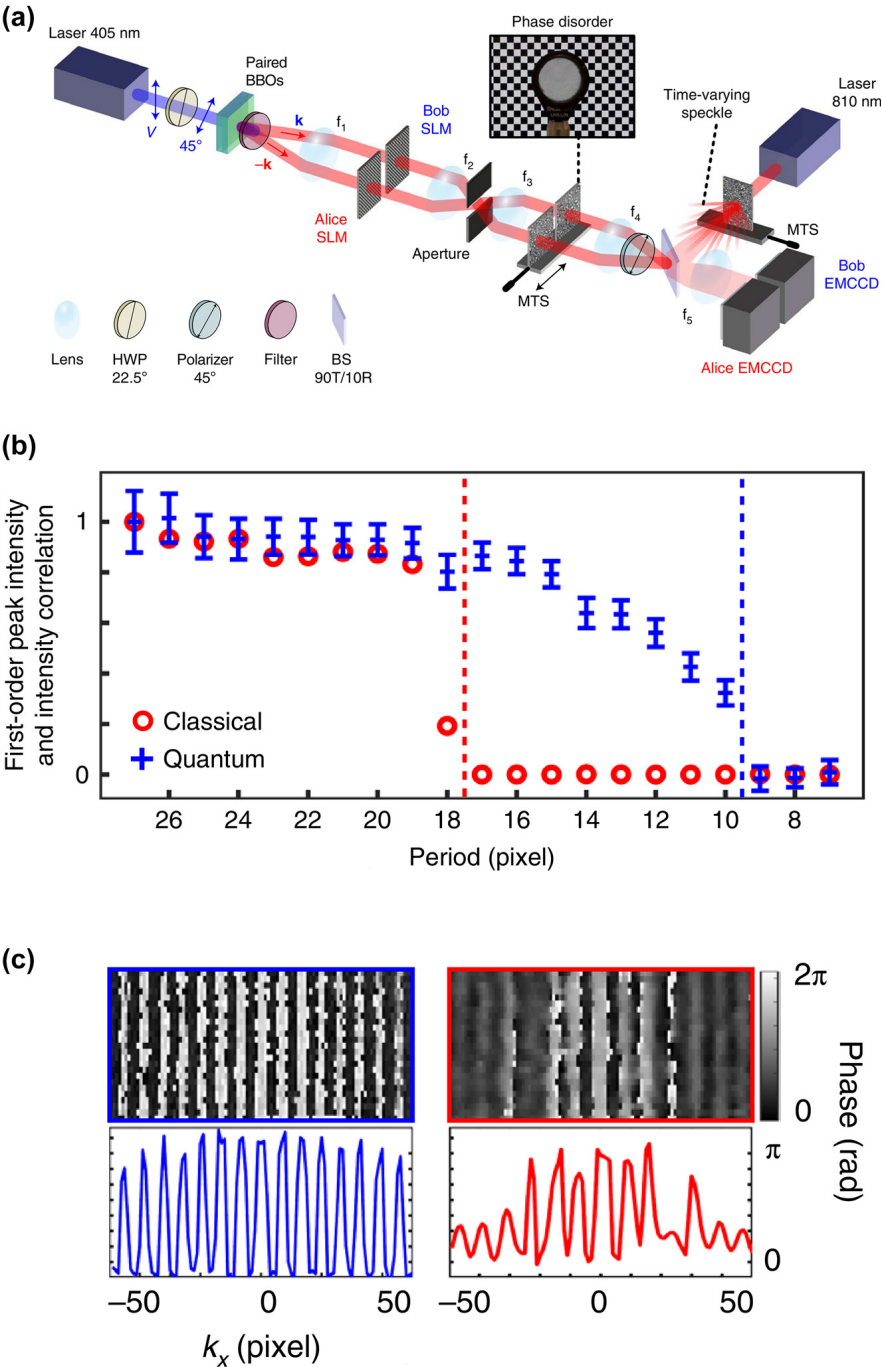
Polarization entanglement-enabled quantum holography. (a) Experimental setup. (b) Measurement of the cutoff period of a grating using classical and quantum imaging methods. (c) Holography of phase grating reconstructed using quantum (left) and classical (right) configurations, respectively [33]. Copyright 2021 Springer Nature.

Another super-resolution method is presented in a study focusing on quantum holography [33]. In Figure 3, the authors propose a wide-field configuration combined with an effective algorithm for $G^{\left(2\right)}$ imaging. The resolution enhancement of the system in Figure 3 is quantified as 1.84, nearly reaching a factor of two. This enhancement factor is derived from analysis presented in Figure 3(b) and (c). The target is a grating generated by the SLM with an adjustable period. The function of the diffraction pattern produced by the grating is similar to that of a slit that is discussed in the theoretical section. The difference is that the Fourier transform of a grating exhibits two distinct peaks. The amplitude of the first-order diffraction peak indicates the capability to resolve the grating periods. The amplitude diminishes as the grating period increases. When the amplitude approaches zero, the cutoff period can be used as an estimate of the system’s resolution, which is similar to the evaluation of modulation transfer function (MTF) [69].

We think that the super-resolution of Ref. [33] has the same mechanisms as QMC. The biphoton source needs to be position-momentum entangled, requiring a multimode source in the imaging system [27], [28]. Because a multimode beam cannot be as finely focused as a single-mode beam, this method has not applied to scanning-based imaging. There have not been any demonstrations of quantum imaging surpassing the best resolution of classical imaging in this manner. One possible reason is that the effective numerical aperture might be less than the labeled one, as the biphoton beam may not fully occupy the apertures of the lenses and objectives.

Here, a statistical algorithm early introduced in Ref. [70] is used to retrieve the image correlation:

(31) $$R\left(I_{s},I_{i}\right)=\frac{1}{N}\overset{N}{\underset{j}{\sum }}\left(I_{s,j}I_{i,j}-I_{s,j}I_{i,j+1}\right).$$

The first term can be interpreted as the second-order correlation function in one frame. As discussed above, this value is skewed by ambiguous events, showing as accidental coincidences. The second term assesses the correlation between adjacent frames, statistically representing accidental coincidences. The true coincidence is estimated to be the subtraction of the total and accidental coincidence. This method is comparable to the covariance algorithm. The difference is the covariance algorithm requires less frame accumulation in the presence of substantial stray light noise.

As a wide-field imaging, optical centroid measurement also achieves super-resolution at the Heisenberg limit. The state representing the center position of an entangled photon pair reads [61]

(32) $$\left|\Psi_{\text{OCM}}\right\rangle =\int d^{2}r_{s}d^{2}r_{i}A\left(\frac{r_{s}+r_{i}}{2}\right)\left|r_{s},r_{i}\right\rangle .$$

Here, *A* is the transmission rate of the object. The centroid coordinate is

(33) $$X=\frac{r_{s}+r_{i}}{2}.$$

We assume the PSFs of the signal beam from the object plane ***r***_*s*_ to the detection plane $r_{s}^{\prime }$ are $h\left(r_{s},r_{s}^{\prime }\right)$ . The same definition applies to the idler beam. The center position is transformed from ***X*** to ***X*** ′ as well; hence, the second-order correlation function is

(34) $$G^{\left(2\right)}={\left|\int d^{2}r_{s}^{\prime }d^{2}r_{i}^{\prime }A\left(X^{\prime }\right)h\left(r_{s},r_{s}^{\prime }\right)h\left(r_{i},r_{i}^{\prime }\right)\right|}^{2}.$$

Then, the correlation function is transformed to the centroid coordinate:

(35) $$\begin{array}{ll} G^{\left(2\right)} & =\left|4\int d^{2}X^{\prime }d^{2}\xi_{s}^{\prime }A\left(X^{\prime }\right)h\left(X+\xi_{s},X^{\prime }+\xi_{s}^{\prime }\right)\right.\\ & \;{\left.\times h\left(X+\xi_{i},X^{\prime }+\xi_{i}^{\prime }\right)\right|}^{2}, \end{array}$$

where ***r***_*s*_ = ***X*** + ***ξ***_*s*_, ***r***_*i*_ = ***X*** + ***ξ***_*i*_, and so on. Specifically, ***ξ***_*i*_ = −***ξ***_*s*_. Present the PSF function as $h\left(X+\xi_{s},X^{\prime }+\xi_{s}^{\prime }\right)\to h\left(X+\xi_{s}\right)$ , the centroid PSF is given by

(36) $$H\left(X\right)=4\int d^{2}\xi_{s}h\left(X+\xi_{s}\right)h\left(X-\xi_{s}\right).$$

Hence, let $\xi_{s}^{\prime \prime }=X+\xi_{s}$ the integral can be changed to a convolution form:

(37) $$H\left(X\right)=4\int d^{2}\xi_{s}^{\prime \prime }h\left(\xi_{s}^{\prime \prime }\right)h\left(2X-\xi_{s}^{\prime \prime }\right).$$

The centroid PSF becomes

(38) $$H\left(X\right)=\left(h*h\right)\left(2X\right).$$

**Figure 4:**
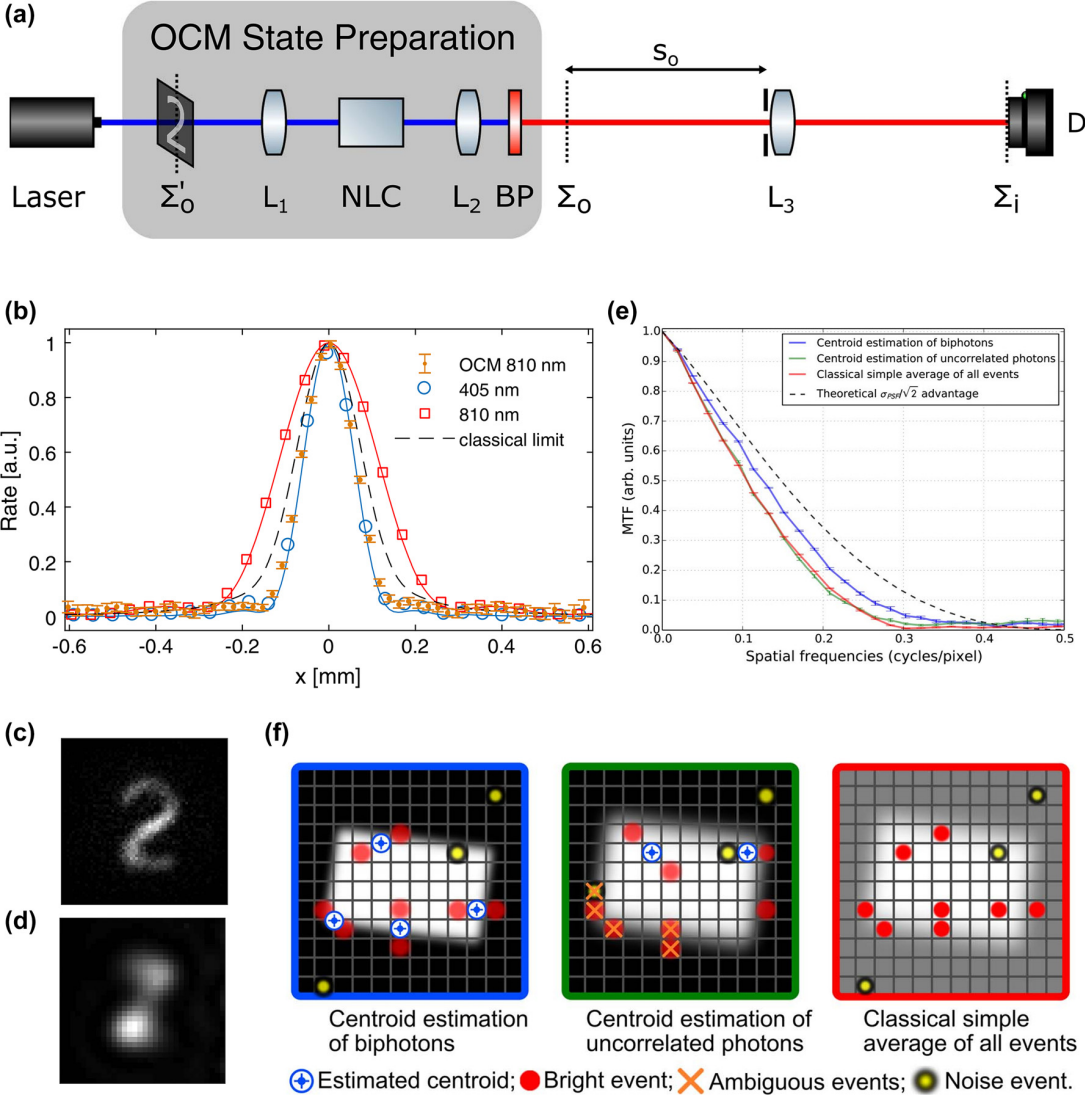
Optical centroid measurement (OCM). (a) Setup of super-resolution at the Heisenberg limit by OCM. Here, the object is placed in front of the biphoton source. (b) The PSFs measured by the setup in (a) with different light sources. (c) Image captured using the biphoton source. (d) Image captured using a spatially coherent laser at 810 nm. (e) Results of a similar setup with the object behind the biphoton source. The slanted-edge MTFs show that resolution enhancement of OCM is 41 % of the anticipated value $1/\sqrt{2}$ . A USAF resolution target was imaged with both biphoton and uncorrelated light sources. The results demonstrated that the blue MTF curve, corresponding to biphoton illumination, displayed a higher cutoff frequency over that corresponding to the red and green curves, which represent classical imaging configurations and uncorrelated photons. (f) Method of centroid measurement [61], [72]. Copyright 2018 Optical Society of America, 2019 Optical Society of America.

In Ref. [61], the authors claimed that, for a single lens PSF jinc function, *h*∗*h* narrows the original PSF *h* by 1/2, which achieves the Heisenberg limit. For a Gaussian pupil function, the centroid PSF should be $1/\sqrt{2}$ narrower than that of the classical beam [71]. This has been observed in Ref. [61], Figure 4(a)–(d) and Ref. [72], Figure 4(e) and (f) as well as other studies using the two-photon NOON state [73], [74]. The resolution enhancement is estimated by the PSFs and MTFs in Figure 4(b) and (e), respectively. The results shown in Figure 4(b) and (e) are measured on different setups where the object was placed at the front and back of the biphoton source. OCM, as a detection method, provides super-resolution in both cases.

Compared to the methods providing two-order resolution enhancement, the resolution enhancement obtained through OCM does not achieve the same limit. For a Gaussian PSF, the enhancement factor achievable by OCM can be similar to that of confocal imaging – a classical method. From the perspective of biphoton imaging mechanisms, OCM reveals a dramatic fact: detecting the center of biphotons yields the same resolution as using the original pump laser, assuming a single-lens configuration.

Theoretically, OCM appears to result from a $G^{\left(2\right)}$ measurement and does not seem to require entanglement. However, the experimental results show that using OCM with uncorrelated light sources may not provide the expected super-resolution. Evidence supporting this is provided by Figure 4(e), where OCM with uncorrelated photons is tested and performs the PSF curve broader than the classical limit. Because the entangled beam generated by a nonlinear crystal is typically multimode, in nonideal situations – where the camera has a large pixel size or the imaging system employs nonideal lenses – this enhancement factor could be even larger due to effects such as entanglement pinhole [65].

### 4.2 Scanning quantum imaging

Spatially entangled photons generated through nonlinear crystals usually have low intensities. Even under ideal conditions for heralding rate and quantum efficiency, the power of a biphoton source remains in the picowatt range. As such, quantum imaging is typically preferred in scenarios that require a low-intensity light field. Furthermore, the FOV in wide-field quantum microscopy is limited by the low light intensity because a larger FOV reduces illumination intensity, resulting in a lower SNR. Quantum imaging employing raster scanning techniques may present a solution to this issue. By focusing the biphoton beam within a scanning microscope, an entangled photon source with increasing coincidence rates can accelerate the imaging process and improve the SNR without compromising the FOV. In raster scanning, however, the FOV is inherently constrained by the scanning range of the mechanical or electronic system [65]. Raster scanning in quantum imaging often entails a trade-off in imaging speed. Since this approach involves sequential scanning of each pixel or point, it tends to be slower than wide-field imaging methods, which can capture the entire sample simultaneously. This limitation is particularly relevant for applications requiring real-time imaging where rapid image acquisition is essential.

**Figure 5:**
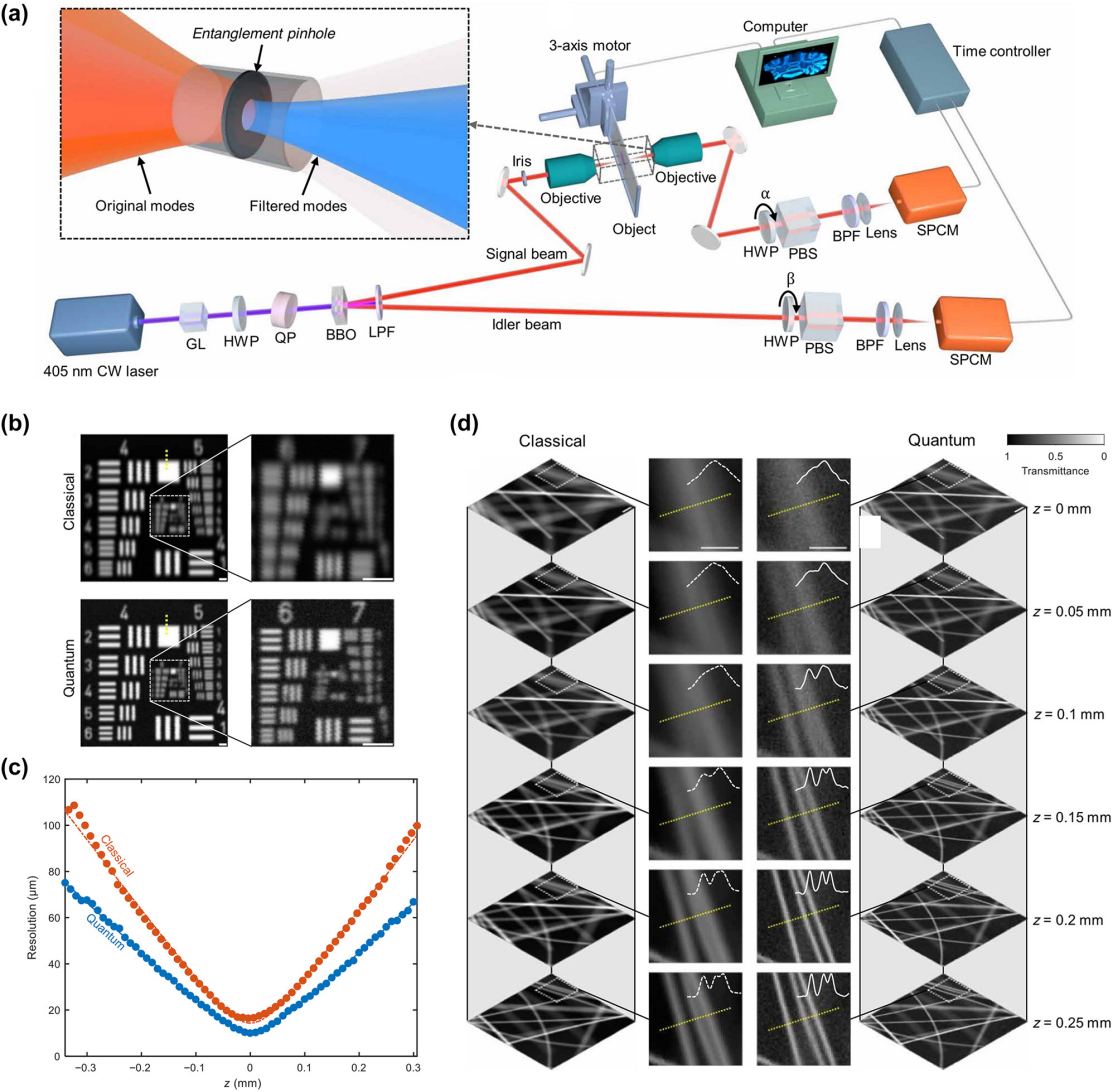
Quantum imaging by coincidence from entanglement (ICE). (a) Experimental setup. Instead of the wide-field configuration, this setup is based on raster scanning. (b) Classical imaging and ICE of a USAF resolution target. (c) Resolution curve for classical imaging (red) and ICE (blue). Dots represent experimental measurements. (d) Classical and ICE images of carbon fibers embedded in agarose at different depths. Profiles along the yellow dotted lines are plotted in the close-ups to compare the spatial resolutions [65]. Copyright 2024 American Association for the Advancement of Science.

Scanning-based quantum imaging techniques, such as imaging by coincidence from entanglement (ICE), are proposed for applications involving polarization entanglement, including birefringence imaging, dark-field imaging, and ghost imaging [65]. In terms of spatial resolution, the results from the scanning configuration, as demonstrated in Figure 5(b)–(d), show that the resolutions of the quantum and classical methods are 14.4 µm and 10.4 µm, respectively. This enhancement intrinsically originates from virtual spatial filtering of the multimode source, known as the entanglement pinhole effect [65]. The resolution enhancement appears analogous to that in the wide-field case, presenting an improvement at various positions along the optical axis. If we assume the single-pixel detector to be ideal, which means the pixel size is much smaller than the beam size, the observed resolution enhancement is primarily due to the multimode focal point being chopped to approach a single-mode focal point. When the biphoton beam reaches single mode, entanglement pinhole effect will not to show further enhancement.

In an extreme case, where the two detectors of the signal and idler photons are smaller than the diffraction limit, the $G^{\left(2\right)}$ is given by

(39) $$G^{\left(2\right)}={\left|\int d^{2}r_{s}^{\prime }d^{2}r_{i}^{\prime }t\left(r\right)h\left(r,r_{s}^{\prime }\right)h\left(r,r_{i}^{\prime }\right)\right|}^{2},$$

where $r_{s}^{\prime }$ and $r_{i}^{\prime }$ are the positions of the detectors; ***r*** is the position of the focal point on the object plane. $t\left(r\right)$ is the transmission amplitude of the object. Here, the PSFs describe the beam propagation from the object plane to the detectors. This creates a comprehensive analogy between the scan-based quantum imaging and confocal microscopy. In this configuration, the two detectors function as the pinholes in a confocal microscope. Consequently, the spatial resolution is primarily determined by the detector sizes as long as they are smaller than the beam size.

## 5 Summary and perspective

The remarkable progress in quantum super-resolution imaging illustrates how early quantum entanglement theory benefits the imaging field while exploring fundamental properties of entanglement. From an imaging perspective, biphoton performance is studied and described as a quasiparticle with half the wavelength of actual photons. The diffraction limits of optical imaging have been reduced by twice, and generating high-order entanglement may further improve the resolution.

The single-mode biphoton source has yet to find its application, but it holds potential for imaging that truly beats classical coherent optical microscopy. Technically, specific methods could be developed for various cameras, revealing potential applications for detectors like superconducting nanowire single photon detectors (SNSPD) and transition edge sensors (TES). Currently, quantum super-resolution imaging mainly supports fundamental research into entanglement mysteries, with future applications anticipated in tasks requiring high sensitivity, high resolution, and low-intensity light fields, such as experiments in cryogenic environments.

The current challenge of the quantum imaging is the low heralding rate of quantum sources generating spatially entangled photons, particularly in nonlinear crystals, and the slow coincidence measurement speed due to statistical reconstruction. These factors affect overall image quality. As for super-resolution microscopy, theoretical predictions suggest that resolution could be further enhanced through multiphoton entanglement. However, quantum imaging has yet failed to achieve an enhancement factor more than two. One significant limit is the complexity of entanglement of more than two photons, coupled with insufficient source power, which reduces imaging quality unless more advanced methods are developed.

Furthermore, the FOV in the wide-field quantum imaging is constrained by the illumination area. Contrary to the illumination provided by a white light source in typical microscopes, quantum sources offer significantly lower power output, generally in a picowatt range [75]. Expanding the FOV thus leads to reduced illumination intensity, affecting the SNRs. Consequently, without advancements in source efficiency, optimizing the tradeoff between the FOV and SNRs remains essential for improving image quality in quantum imaging. So far, although the biphoton state offers super-resolution, in most experiments, the low SNRs, small FOVs, and low throughput make this technique difficult to use and align.

In recent years, there have been developments of novel types of quantum sources and detectors. For instance, ultrabright on-chip quantum sources have been developed and reported primarily for applications in quantum communications and computing [76]. High-power sources hold the potential to enhance quantum imaging by improving the SNRs and speed, thus broadening their application to imaging tasks requiring classical sources. Additionally, single-photon avalanche diode (SPAD) arrays are being employed in cameras for quantum imaging [77]. This integration of single-photon detection with two-dimensional sensing has yielded promising results [36], [78]. Early SPAD arrays had limited pixel numbers and large pixel sizes, severely restricting the FOV and spatial resolution. Recent advancements have seen SPAD cameras with over 512 × 512 pixels, addressing previous limitations [79]. However, the quantum efficiency of SPADs still falls short compared to EMCCDs operated at low temperatures, posing future challenges for their usage. Looking ahead, the integration of novel sources and detectors is expected to drive further advancements in quantum super-resolution imaging.
